# Strategies to customize responsible gambling messages: a review and focus group study

**DOI:** 10.1186/s12889-018-6281-0

**Published:** 2018-12-17

**Authors:** Sally M. Gainsbury, Brett L. L. Abarbanel, Kahlil S. Philander, Jeffrey V. Butler

**Affiliations:** 1 Brain and Mind Centre, School of Psychology, University of Sydney, Camperdown, NSW Australia; 2 International Gaming Institute, University of Nevada, Las Vegas, Box 456037, 4505 S. Maryland Pkwy, Las Vegas, NV 89154-6037 USA; 3 UCLA Gambling Studies Program, University of California, Los Angeles, USA; 4 School of Hospitality Business Management, Carson College of Business, Washington State University, Everett, WA USA; 5 Department of Economics, School of Social Sciences, Humanities and Arts, University of California, Merced, Merced, CA USA

**Keywords:** Customized messaging, Targeting, Prevention messages, Problem gambling, Responsible gambling, Education, Harm minimization, Youth, Seniors

## Abstract

**Background:**

Responsible gambling messages are widely used as a tool to enable informed choice and encourage appropriate gambling behavior. It is generally accepted that gamblers have different levels of risk of developing gambling problems and require various harm minimization tools and resources. Therefore, it is reasonable to expect that responsible gambling messages should be customized and target specific groups of gamblers. This project aimed to understand hypothesized differences between cohorts of gamblers and receive qualitative feedback on archetypal targeted messages used to increase use of responsible gambling tools.

**Methods:**

Focus groups were held to test messages for specific cohorts: young adults (18–24 years), seniors (60+ years), frequent gamblers (weekly), and gamblers of skill-based games (poker, sports betting).

**Results:**

Cohorts exhibited different preferences and responses to message archetypes. Seniors preferred messages about limit setting, whilst young adults and frequent gamblers responded to messages about their own play and expertise. Skill game gamblers were interested in the odds of winning and their own outcomes over time. However, all groups agreed that using positive, non-judgmental language in messaging is important.

**Conclusions:**

This research makes an important contribution to the field by demonstrating that the wording of message content will likely influence the effectiveness of such messages differentially across various groups of gamblers for engaging gamblers in harm reduction tools. Guidance is provided on themes that can be used by public health marketers.

**Electronic supplementary material:**

The online version of this article (10.1186/s12889-018-6281-0) contains supplementary material, which is available to authorized users.

## Background

Many wellness oriented interventions are available for gamblers. Across jurisdictions, programs may include psychosocial treatment, awareness campaigns, player education programs, self-exclusion programs, and play management resources, such as limit-setting tools. But whilst programs are often available, there are typically barriers to help-seeking for those who would benefit from their use. These issues include stigma, shame, lack of knowledge, unwillingness to admit a problem, and/or wishing to handle a problem by oneself [1–3]. Effective interventions are important to assist gamblers at various levels of risk to acquire and apply the requisite skills and knowledge necessary to control their gambling to affordable levels. Encouraging gamblers to seek help before their problems become severe would minimize harm, as well as reduce the burden on emergency and treatment services. However, individuals who engage in risky gambling but are not experiencing serious problems may lack motivation to seek help proactively and appear to be relatively hard to reach.

Given that modern player tracking systems can now display user-specific information on electronic gambling machine screens or personal electronic devices, there has been substantial interest within the academic and responsible gambling (RG) community in using customized RG messages to improve informed decision making [4]*.* Prevention messages are one of the most widely used public health strategies for reducing harms from gambling. Messaging seeks to elicit direct changes in behaviors/beliefs and may also inform the public of associated risks or RG programs. With recent technological changes in gambling products, institutions responsible for executing RG programs have hypothesized that customized messages may be more effective than a one-size-fits-all messaging program. RG messaging is typically a broad-spectrum strategy that is often the first-point of contact with gamblers. Even small improvements in the effectiveness of communicating such messages may have large downstream impacts on harm.

In this paper, we explore the use of customized messages targeting various segments of gamblers. Messages are framed to increase gamblers’ motivation to seek and use relevant RG resources. As part of this study, we review the broader public health messaging literature for its relevance to RG messages. We examine whether the themes emerging from the broader literature also manifest in the content of focus group discussions within a community sample of gamblers regarding a series of RG messages presented to them. We make an academic contribution to the public health messaging literature by framing categories by which gambling messages could be customized in order to facilitate engagement with harm minimization resources. We also make an applied contribution by exploring message archetypes which, after adaptation to regional idiosyncrasies, could be adopted by prevention programs within a cohort of end-users.

### Responsible gambling

Gambling disorder is an addictive disorder described as, “persistent and recurrent problematic gambling behavior leading to clinically significant impairment or distress” [5]. The disorder is characterized by a range of symptoms, including distorted cognitions, chasing losses, preoccupation with gambling, and inability to stop [2, 3, 6]. Research indicates that gamblers generally support the availability of RG tools, particularly those that assist customers to play within their means, including player feedback and regular financial statements [7–12]. Despite that, customer engagement with RG tools appears to be relatively low.

Broadly, uptake of RG resources has been low. In a recent British Columbia study, Cohen, McCormick, and Davies [13] estimated that only 5% of the moderate to high-risk population are enrolled in the province’s self-exclusion program. Within Australia, one site reported that only 1600 out of 200,000 active customers (0.8%) used the deposit limit tool available, and only 900 self-excluded from the site (0.45%) [14]. Reports from a European online site indicated that only 1.2% of users self-imposed limits on their expenditure [15], while another European site found only 13% of users regularly engaged with a well-integrated RG tool [16]. Research on limit-setting tools suggests that the vast majority of gamblers respond positively to the concept of pre-commitment, but need direction to make use of such tools [17–19].

Research from the broader health messaging literature, relating to sexual health, alcohol consumption, and nutrition behaviors, suggests that messages could have increased effectiveness for such populations by communicating direct and tailored content (e.g., RG tips), rather than simply providing information about the availability of programs and resources [20–22]. Critical to the effectiveness of such messages are the type of content used, the way they are framed, whether they engage consumers in self-referential processing, their level of specificity and applicability to use in the real world, and the use of social norms to influence the behavior of the individual. Findings on the importance of these factors from the broader public health messaging literature are now reviewed in order to consider their relevance to RG messaging.

### Message content

Important elements of consumer communication include language, tonality, and message content [23, 24]. Message content refers to the simplicity, degree of directness, and comprehension of the words that communicate the appropriate level of danger, consequences, and/or actions to avoid harm [25]. The type of language used in warning messages may have varying impacts on individuals, depending on culture, emotional state, level of gambling problem, and the consumer’s sense of self-esteem [26–28].

RG messages often inform gamblers of information about the probabilities of winning, and how outcomes are determined. These messages are predicated on the use of warnings for alcohol and tobacco products, informing consumers about the risks associated with excessive or inappropriate use. In the gambling context, the use of informative or educational messages is based on the concept of problem gambling being a result of irrational thoughts and beliefs. It was hypothesized that if gamblers understood the games and probabilities of winning, they would be able to make informed decisions regarding their involvement [29–31]. Empirical research suggests that effectively communicated information does not consistently modify irrational beliefs or erroneous estimations about the chances of winning [32–36].

The failure of such information to modify behavior is likely due to cognitive biases that enable gamblers to understand the low probabilities of winning, yet still believe that they may have a chance to win [36]. Even when informative messages are accurately recalled, respondents still believe that their chances of winning are greater than the information contained within the messages, and do not modify their behavior [37, 38]. Whilst informative messages can correct irrational beliefs, there is limited empirical understanding of how such messages may impact gambling behavior [35, 36].

### Message framing

Prospect theory suggests that people behave differently when messages are framed as either gains or losses [39]. Positive or gain-framed messages focus on the benefits of making improvements in a particular behavior, whilst negative or loss-framed messages contain information about harmful consequences and hazards related to risky behaviors [27]. Although educating consumers about the risks of a product is connected to informed choice, research on attitudes and persuasion suggests a focus on negative impacts is too narrow [40], and a gambling limit tool study showed that feedback about violating a limit did not lead to the intended reduction in play [41]. Messages are more persuasive if they promote positive attitudes, and if the identified behaviors are mutually exclusive (e.g., setting deposit limits vs. having no limits) [42]. The use of positive framing of messages has been found to have a greater impact than negative framing. For example, neuroimaging research has found that gain-framed messages are more effective in improving risky choice behaviors than loss-framed messages amongst individuals with substance-use disorders [43, 44]. When a behavior is presented as a choice, positive messages are more effective than negative messages [45]. Audiences tend to respond with more enthusiasm to positive things that they can do to prevent health problems [46], and research across multiple public health domains suggests that positively-framed messages are more effective than loss-framed messages when advocating health prevention behavior [46].

### Self-appraisal messages

Self-appraisal messages encourage consumers to reflect on their own personal situation and take appropriate actions. Persuasion research shows that when individuals generate arguments and conclusions themselves, they are more convincing for the individual than statements provided by external sources [47]. Self-generated arguments are often perceived as more accurate than information provided by external sources [48–50]. Messages that imply an outcome but allow perceivers to draw their own conclusions may reduce feelings of resentment and enhance the persuasiveness of messages [51]. Several laboratory and in-venue trials of self-appraisal messages on electronic gaming machines (e.g., “Have you spent more than you intended?”) have found that such messages increase awareness of time spent playing and create more realistic thoughts about the chance of winning, increasing the likelihood of taking a break and reducing the duration of gambling sessions [38, 52].

### Specific and action focused

Less abstract messages that include specific actions, such as setting a deposit limit, can increase message compliance [53]. Research with smokers found that warning messages should contain sufficient information and identify steps to help smokers progress towards quitting [54–56]. Online gambling messages that suggested specific information (e.g., “10 gambling commandments”) generated five times more website “click-throughs” than informative messages commonly used (e.g., “How problem gambling works”) [57]. A sense of urgency can also be introduced by using phrases such as, “Have you… yet?”. This is consistent with research on health warnings which has demonstrated that messages that are positive and have a sense of urgency are felt to be strong motivators for action [58].

### Targeted messaging for gamblers

Attempts to warn players of risks associated with gambling and direct them to RG resources often use in-venue signs with RG slogans and problem gambling helpline numbers. Many studies have found that these messages are largely ignored by gamblers [38, 59, 60]. The extent to which the message is read, absorbed, and acted upon is dependent upon the personal relevance of the message, the target recipient’s capacity to assimilate the information, and their motivation to respond [25]. To be effective, RG messages should engage the gambler’s cognitive, emotional, and motivational faculties, and alter the behaviors of concern [61–63]. It is plausibly unreasonable to expect that messages broadcast to all gamblers can be impactful given the many differences between players, including the type of resources they would each benefit from using.

Whilst legacy gambling systems were more inflexible, new technology linking player accounts can enable sophisticated RG strategies, including personalized messaging that targets players based on individual characteristics and patterns of play [64]. For example, gambling providers can send direct emails, online messages, or SMS to customers. Additionally, within venues where loyalty cards are used, operators can send personalized RG messages to customers through electronic gaming machine screens. Tailored messaging has been shown to outperform traditional, static health information strategies, and is more likely to be read, remembered, and viewed as personally relevant [65]. Importantly, tailored messaging has been shown to be important in motivating change in problem drinkers and problem gamblers, irrespective of whether they commence treatment or not [66, 67].

### Segmenting gambling cohorts

Targeted messages should be particularly useful in populations where there is great variability between members [68] and that information is accessible in marketing databases. One key difference between gamblers is age. Young adults (aged 18–24) appear to manifest more gambling-related problems [69–71]. The reception of warning messages by young adults is often considered to be different from the general population [24, 72]. In their meta-analysis of evidence on warning message effectiveness, Argo and Main [23] argue that age correlates negatively with warning perception, although they note relatively limited empirical evidence to support this. Young adults tend to perceive themselves as invulnerable to the negative consequences of risky behaviors, and have difficulty relating to negative consequences that may occur in the future [73–76]. Young people also tend to underestimate the severity of their gambling, fail to recognize and accept gambling problems, and are less likely to seek help [77]. They do not necessarily have a poorer understanding of gambling odds than adults, but they are more prone to erroneous beliefs about gambling, as well as beliefs that gambling can be controlled [78–80]. Due to the increased relevance of social norms for youth, manipulating social context may increase the effectiveness of messages for this target cohort [81]. For example, smoking and drug prevention advertisements highlighting social implications appear to be more persuasive than warnings of physiological illnesses with adolescents and young adults [82, 83].

In contrast, older adults are another group with unique risks and importance. According to the 2014 Canadian Community Health Survey [84], over 67% of persons aged 65 or older gamble. Many scholars have emphasized the importance of protecting this group from the harms of gambling, noting the risk factors associated with fixed-incomes, social isolation, bereavement, and increased leisure time in retirement [85–87]. One key difference between seniors and other groups is that they show more obsessive passion for gambling when their behavior is problematic [88]. They also show a greater likelihood of responding to digital marketing strategies than other sub-groups [89].

It is also plausible that different gambling product users will receive messages differently. Gamblers who engage in games with real or perceived skill (e.g., poker, sports betting) view themselves as different, as they use their ability to increase their odds of winning [90, 91]. Online poker players are generally less likely to chase losses than online casino gamblers [92], respond better to time limit tools than monetary limit-setting tools (unlike other gamblers [93]), and are a heterogenous group in that more skillful online poker players have been shown to respond less favorably to RG tools less skilled players [7].

Frequent gamblers – usually characterized by participation in gambling once a week or more – have been identified as having greater risk for gambling problems. Research has shown that frequency of participation is a highly predictive risk-factor for gambling-related problems [94–96], and may be a behavioral marker for gambling disorder [97, 98]. Given the specific characteristics of this group, frequent gamblers appear to be another plausible target group to be studied for tailored RG messaging, alongside young adults, seniors, and skill game gamblers.

There are several variables that can be considered in designing and distributing RG messages, and message impact is likely to differ between various segments of gamblers due to their own needs and preferences. There appears to be adequate evidence in the gambling literature to warrant customization of messages for young adults, older adults, skill-game gamblers, and frequent gamblers. While other risk groups exist, we examine these categories as they can be identified in most marketing databases. Age is collected as part of the entry control requirements in the registration processes, while game type and frequency are variables controlled by the operator. In the next section, the approach to exploring thematic differences in these cohorts’ needs is described.

## Methods

A series of online focus groups were conducted to gain feedback on the wording of various RG messages created based on the literature review for each of four selected player cohorts. The study protocol was reviewed and approved by the Human Subjects Ethical Review Committee at Southern Cross University. Focus groups were used because they are socially-oriented, in which participants listen to others’ opinions and understandings in forming their own responses [99]. Whilst focus groups typically have high face validity, group size may be small and the results may not be statistically generalizable [99, 100]. The results, however, still provide meaningful insights into how participants respond to messages presented to them, as well as feedback on message wording and content. As any message used in actual marketing materials would need to be adapted for its particular market, focus groups are a useful method for exploring themes that a generic strategy could consider.

The four player cohorts were identified based on a review of the literature and are groups that could be easily identified in player databases for message distribution. In total, 39 participants attended the four focus groups:

1. *Young Adults* – those aged 18–24 years old (*N* = 10, 6 male)
2. *Seniors* – those aged 60 years old or older (*N* = 10, 4 male)
3. *Skill Game Gamblers* – those who play games involving an element of skill, such as poker or sports betting (no age qualification for this group) (*N* = 10, 7 male)
4. *Frequent Gamblers* – those who have gambled once per week or more often (no age qualification for this group) (*N* = 9, 6 male)

Each of the authors independently generated potential messages for the cohorts. That list of potential messages was then reviewed by all authors for content and relevance within the literature framework and reduced to a useful subset through consensus. Messages were selected to be similar to archetypal messages used by RG programs, but also with consideration of the evidence for their effectiveness found in the health messaging literature review. Messages were then reviewed for appropriate length before inclusion in the focus group moderator guide, which can be viewed as supplementary file Additional file 1.

Each message contained an action phrase connected to an RG tool to prompt respondents to consider their behavioral response. The Play Summary tool displays the player’s play history. The Player Assessment Quiz is an online quiz that helps gamblers better understand their own gambling behavior within the scope of health habits. The Responsible Gambling Tips are seven tips that encourage players to gamble responsibly, such as “Don’t chase losses,” and “Take frequent breaks”. The Odds Knowledge Quiz is an online quiz that tests the participant’s knowledge of gambling odds, such as the roulette wheel landing on a given number. The Limit Setting tool is a budgeting tool that allows players to set a weekly limit on the total amount they can transfer into their online gambling account. Table 1 below displays the messages presented to the respective groups.

**Table 1:** Messages Presented to Each Focus Group, by RG Tool

	RG Tool					
Group	Play Summary	Player Assessment Quiz	Responsible Gambling Tips	Odds Knowledge Quiz	Limit Setting (a)	Limit Setting (b)
Young Adults	Do you know how much you are spending? Check your Play Summary.	What kind of player are you? Take this short quiz.	Play often? Check out these 7 gambling tips.	Are you a gambling expert? Test your knowledge of gambling odds.	All players need to stick to their limits. Have you set your spend limit?	All players need to stick to their limits. Do you know how much you have spent?
Seniors	Have you spent more than you can afford? Check your Play Summary.	What kind of player are you? Take this short quiz.	Play often? Check out these 7 gambling tips.	Are you a gambling expert? Test your knowledge of gambling odds.	All players need to stick to their limits. Have you set your spend limit?	All players need to stick to their limits. Do you know how much you have spent?
Skill Game Gamblers	Do you know how much you are spending? Check your Play Summary.	What kind of player are you? Take this short quiz.	Play often? Check out these 7 gambling tips.	Are you a gambling expert? Test your knowledge of gambling odds.	All players need to stick to their limits. Have you set your spend limit?	All players need to stick to their limits. Do you know how much you have spent?
Frequent Gamblers	Have you spent more than you can afford? Check your Play Summary.	What kind of player are you? Take this short quiz.	Play often? Check out these 7 gambling tips.	Are you a gambling expert? Test your knowledge of gambling odds.	All players need to stick to their limits. Have you set your spend limit?	Even frequent players need to stick to their limits. Do you know how much you have spent?

Included with each message is a link to the respective RG tool

### Participant recruitment

Participants were selected and screened by telephone using a database comprised of randomly selected households in Manitoba, Canada. Participants qualified if they were over the age of 18 and had reported gambling online during the prior 12 months, to ensure the groups were formed with participants who were potential recipients of these types of messages. Table 2 below displays core sample characteristics reported during the screening process, including gender, age, gambling activity, and gambling frequency, which was measured as a simplified dichotomous variable for screening into the frequent gamblers group. During the recruitment and screening process, all participants completed an informed consent form. All participants were provided with a $50 incentive for their participation.

**Table 2:** Sample characteristics (*N* = 39)

		Percent
Gender	Male	59.0
	Female	41.0
Age	18–24 Years	30.8
	25–40 Years	17.9
	41–59 Years	25.6
	60 Years and older	25.6
Gambling Activity Online, within Prior 12 Months	Poker, Blackjack, and other skill games	46.2
	Sports Betting (including fantasy leagues)	51.3
	Bingo	10.3
	Lotteries	56.4
	Chance-based table games (e.g., roulette or craps)	5.1
	Slots	38.5
Gambling Frequency	Once per week or more often	43.2
	Less often than once per week	56.8

### Data collection

The four online focus groups ranged from 60 to 78 min in duration and were conducted via iTracks, an online focus group platform. Research suggests online focus groups may elicit more themes with sensitive topics than in-person focus groups [101]. The iTracks platform conducts focus groups in a written format, similar to an online chat room. The online focus group was conducted in a similar manner to an in-person focus group, with a moderator leading a discussion among a small group of participants. The participants submitted typed answers to the group, and the moderator was able to communicate privately with individual participants. Through the private messaging system, the moderator was able to ask individual follow-up questions whilst continuing the discussion in the main focus group.

Focus groups were structured to elicit responses to the proposed messages, with an interest in collecting participant feedback on six pre-developed messages per group. The messages were presented in five cases, one at a time; each case focusing on a different RG tool (as displayed in Table 1). Participants were first asked for their initial response to the message, and then asked why they would or would not engage with the linked tool. Next, the inquiry requested any proposed changes that participants would make to the message to make it more relevant to them. Where relevant, the moderator would send follow-up messages to individual participants to elicit further details on their response to the individual messages. Finally, participants discussed the types of messages they found most effective, and offered up their own wording, themes, or specific phrases that would get them to engage with RG tools.

### Analysis

The transcriptions of the focus groups were subjected to content analysis with a focused coding approach. Content analysis was used because it provides a “careful, detailed, systematic examination of a particular body of material in order to identify patterns, themes, biases, and meanings” [102]. In focused coding, researchers identify themes and look for associated data fitting under categories of interest [103]. Coding categories were established based on the literature review and focused on message tone, potential engagement, personalization options, numbers, and terminology (e.g., gambling vs. gaming, player vs. gambler). The *message tone* theme was used to assess participant interpretation of the tone of the message (e.g., encouraging, condescending), and their emotional response (positive, negative, neutral) to that tone. The potential engagement category was used to code how participants indicated they would or would not engage with the RG tool associated with the message. The personalization options theme covered participant suggestions of how the messages could be individualized for different people or player types. The numbers theme described inclusion of numbers from individual play behaviors, such as amount of time or money spent gambling. Finally, the terminology code was used to identify where participants identified qualities of the vocabulary used.

To establish inter-rater reliability, two researchers independently coded the data set (98.6% agreement across 76 entries), then together reviewed and settled the only discrepancy in coding by considering its content, theme definitions, and extant literature support.

## Results

The messages recommended for each specific group as a result of the analysis is presented in Table 3. Within all groups, there was no difference in message preference between genders.

**Table 3:** Recommended messages for Young Adults, Seniors, Skill Game Gamblers, and Frequent Gambler group types

Group	Generic	Message 1	Message 2
Young Adults	Only spend what you can afford to lose. Check out the play management tools.	Are you a gambling expert? Test your knowledge of gambling odds.	Keep it a game. Check out these 7 tips to become a more responsible gambler.
Seniors	Only spend what you can afford to lose. Check out the play management tools.	How much have you spent gambling? Check out your play summary here.	Stick to your limits and keep gambling fun. Have you set a spending limit?
Skill Game Gamblers	Only spend what you can afford to lose. Check out the play management tools.	Check out your gambling odds. Test your game knowledge here.	Check out your play summary. Click here to see your spending habits.
Frequent Gamblers	Only spend what you can afford to lose. Check out the play management tools.	What kind of gambler are you? Take this short assessment quiz here.	Even frequent players should have limits. Have you set your spend limit?

Included with each message is a link to the respective RG tool

### Young adults

Young Adults were particularly responsive to message tone, especially messages that were perceived as condescending. One participant requested messaging that “does not sound like it is ‘blaming you,’” and several others followed along the same train of thought, asking for messages that were not accusatory or patronizing, but rather were straightforward and honest in their phrasing. Simple messages were preferred, as one participant explained, and not “dressed up in language.”

Young Adults also indicated a preference for messaging that provided tips to show how they can save money, by using the linked RG skills and tools. Some follow-up comments suggested that participants were interested in messages with tips that helped them become more successful gamblers (i.e., to win more money). Other tools that drew a positive response from Young Adults included the Play Summary tool, which was identified as a useful tool for accurately recording play when gamblers’ own perception might be distorted, and the quiz testing knowledge of gambling odds, which was labeled as “beneficial.” Conversely, the Young Adult group indicated a distinct lack of interest in the self-assessment quiz, which they viewed as not useful.

The idea of presenting negative realities was also suggested – the discussion turned to the potential harms of problem gambling and a proposal for warning messages similar to those on cigarette packs was presented as an option for messaging.

### Older adults

Like the Young Adults, Older Adults were also concerned about tone, with one participant stating that they wanted messages that “treats [one] like a responsible adult,” and not like “my mother wagging her finger at me.” Other participants suggested avoiding “condescending messages,” and that it felt “insulting” to “imply that [they are] foolish to not set a limit”, when provided a message on limit-setting tools. Beyond this concern, the group was positive about the use of messages in general, suggesting that they be clever, upbeat, and humorous, with reminders to keep the game fun. Some participants suggested that additional messages that show negative consequences of problem gambling would also be useful, as well as information on where to get help if a gambler thinks they are losing control.

Older Adults identified the Play Summary as a useful tool, though many indicated they already set limits when they play. In addition, the quiz to test gambling knowledge and the limit setting tools were selected as tools that the group would seek out if they received a message promoting the tool.

### Skill game gamblers

Skill Game Gamblers were largely interested in blunt, straightforward language in their messages, with one participant requesting messages that “[call] a spade a spade.” Messages that were simple and direct were preferred, such as those that included specific values for time and money spent gambling. Matching this preference, Skill Game Gamblers indicated that the Play Summary was a valuable tool for them, making them think about their own budget. The group also requested individualized messages with their personal spend numbers, as “seeing real numbers tells the story.”

The quiz testing gambling knowledge was also considered useful, primarily for the purpose of confirming their own knowledge. One participant also indicated that a message promoting this kind of quiz would get them to engage with the tool to enhance their knowledge of responsible gambling, stating, “if I could learn something that could help me place a responsible bet, I’d read on.”

Skill Game Gamblers also suggested that messages should include reminders of the ramifications of overspending, as well as that the odds are against winning and that in the long run, the house always wins. In addition, Skill Game Gamblers were the only group to speak to terminology use in describing those who gamble, suggesting that use of the terms “gamble” or “gambler” instead of “play” or “player” would be useful reminders that wagers involve at least some chance component.

### Frequent gamblers

As with the other groups, Frequent Gamblers emphasized the need for positive language in RG messaging, and to avoid any language that might be accusatory or might make someone feel guilty about their gambling behavior. Simple, short messages were preferred, such as reminders to “keep it a game.” One participant further recommended messaging include information to educate family and friends who might need the RG tools. Individual spend numbers were requested as a reminder of play behaviors, paralleling the request of Skill Game Gamblers.

Frequent Gamblers were the only group to not positively endorse the Play Summary tool, with most participants indicating they were already aware of their limits and spend, and thus felt they did not need the tool. Also, distinct from other groups, Frequent Gamblers responded positively to the self-assessment tool, expressing curiosity about their classification. Several participants noted that they “love taking short quizzes” and that they were interested into which player type they would fall.

## Discussion

This research is the first study to empirically consider the content of RG messages in identifiable cohorts. In this study, thematic differences were found between subgroups of players. This was observed in both message wording (e.g., tone) and in the action response (i.e., RG tool) that they were prompted to consider. Young Adults gravitated towards tips to help them be ‘better’ gamblers, which could be used to provide suggestions for losing less money (e.g., not chasing losses). This is consistent with research suggesting that young people are more prone to erroneous beliefs about gambling, such as the idea that gambling can be controlled [78–80]. The focus group supported this, with participants reporting that they would find the information in the quiz and list “beneficial,” and that they would use the tools to “gain a greater understanding of what [they’re] looking at” when they’re placing bets. In contrast, Older Adults looked for more light-hearted messages, focused on keeping gambling fun. Older Adults were also attracted to limit-setting features that were not as popular among other groups. Older Adults, many of whom are on a fixed income, may benefit from tools that help them be mindful of how much money they spend on gambling [104, 105].

The self-assessment test of gambling behaviors and limit setting tools were viewed more positively by Frequent Gamblers. Frequency of gambling has been identified as predictive of problem gambling [106], and these messages prompt the recipient to assess their own level and style of gambling, and also suggest a tool that can be used to help them keep their gambling within affordable levels [105].

Skill Game Gamblers preferred more direct communication, seeing themselves as able to incorporate information into their gambling, including about potential risks of gambling. Frequent Gamblers were interested in resources to assist them in keeping track of their expenditure – such as activity statements in the Play Summary tool. This is consistent with Philander and Gainsbury [105] who suggest that because those who play games with an element of skill are more likely to develop illusions of control about their skill [107], messages for these gamblers should encourage gamblers to be mindful of the element of chance in games. The Play Summary message also serves as a reminder to Skill Game Gamblers to be mindful of their sessions and expenditure. Recreational Skill Game Gamblers can exhibit impulsive personality traits and may demonstrate signs of chasing behaviors [108–110], and this message may encourage them to use the Play Summary tool as a means of keeping themselves aware of expenditure resulting from such behavior.

Messages were found to be more likely to be persuasive if they promote positive attitudes towards the desired behavior. Motivation can be enhanced by reducing the ‘cost’ of compliance, increasing the perceived ability to perform a specific action, using a positively-framed message, and appealing to the individual’s sense of value. Gamblers may be more likely to engage with RG resources if they believe that these resources are typically used by their peers, and those that they respect. Focus group participants consistently discussed the importance of messages not being patronizing or judgmental.

Educational- and awareness-based messaging is a tool that the gambling field has adopted from the wider public health field, with mixed success. Customizing RG messages and pairing RG tools based on age, gambling frequency, and type of gambling activities may enhance the effectiveness of messages and subsequent engagement with RG resources. Literature supports the use of messages that encourage gamblers to consider their own gambling, rather than providing explicit directions or information; a finding reinforced by participants in this study. When individuals generate arguments and conclusions themselves, they are more convinced than by statements provided from external sources.

### Limitations & future research

Whilst focus groups are highly useful for the in-depth exploration of topics, attitudes, and concerns, the findings may not be generalizable due to the limited sample. In addition, with only one group per segment type, the ability to draw conclusions from the data is limited, as data saturation may not have been reached. Third-person bias may be at play, where messages that focus group participants think will be persuasive may be influenced by participants considering what is effective for other people, rather than themselves. The results are also limited in that they only considered four cohorts, each with some degree of overlap. It is likely that gambling operators can segment their player databases in more sophisticated manners.

Further research should aim to analyze player databases of gamblers to identify at-risk gamblers using more complex segmentation, connected to a theoretical understanding of persuasive messages and experimental design. Such an approach could include qualitative research more specific to the given jurisdiction, followed by field experiments testing multiple message options. Future work should also consider how to deliver messages, in terms of mode of delivery, frequency, and duration. Each of these factors is important to effective communication.

Last, it is important to note that gambler preferences are not the only consideration in the design of public health strategies. Although gamblers may prefer one RG tool to another, that does not mean that this is the tool from which they would most likely benefit [111]. As such, one important role for RG messages and public health communication strategies is to effectively describe available resources to enhance understanding among relevant groups and evaluate related outcomes, even where there is not intrinsic interest in their use.

## Conclusions

By focusing messages on a specific intended audience, messages can be developed to elicit greater individual responsiveness and compliance. Our research suggests that there are some commonalities in message components that are perceived to be most effective in encouraging uptake of preventative and harm-minimizing behaviors. This includes promoting positive attitudes towards the desired behavior and reducing the perceived cost of compliance – that is, positive framing, and making behaviors easier and simpler to complete. Increasing specificity of messages also enhances engagement, particularly if a sense of urgency is conveyed. Personalizing messages to target specific population subgroups and understanding the characteristics of those subgroups is advantageous and likely to enhance the presentation of health information.

## Additional file

Additional file 1: Focus Group Moderator Guide for Strategies to Customize Responsible Gambling Messages: A Review and Focus Group Study. Moderator guide information for focus groups conducted, including research background, initial questions, and proposed RG messages for testing. (PDF 112 kb)

## Abbreviation

RG: Responsible Gambling

## References

[1] Hing N, Nuske E, Gainsbury S, Russell AMT (2016). Perceived stigma and self-stigma of problem gambling: perspectives of people with gambling problems. Int Gambl Stud.

[2] Kim HS, Wohl MJ, Salmon M, Santesso D (2017). When do gamblers help themselves? Self-discontinuity increases self-directed change over time. Addict Behav.

[3] Suurvali H, Cordingley J, Hodgins DC, Cunningham J (2009). Barriers to seeking help for gambling problems: a review of the empirical literature. J Gambl Stud.

[4] Langham E, Rockloff M, Browne M, Best T (2017). Could EGM player-tracking systems help link gamblers to treatment services in Australia: a thematic analysis of counsellor and community educators’ perspectives. Int Gambl Stud.

[5] Diagnostic and statistical manual of mental disorders (DSM-5®) (2013). Washington.

[6] Hodgins DC, Stea JN, Grant JE (2011). Gambling disorders. Lancet.

[7] Gainsbury S, Parke J, Suhonen N (2013). Consumer attitudes towards internet gambling: perceptions of responsible gambling policies, consumer protection, and regulation of online gambling sites. Comput Human Behav.

[8] Gainsbury S, Russell A, Blaszczynski A, Hing N (2015). Greater involvement and diversity of internet gambling as a risk factor for problem gambling. Eur J Pub Health.

[9] Griffiths MD, Wood RT, Parke J (2009). Social responsibility tools in online gambling: a survey of attitudes and behavior among internet gamblers. Cyberpsychology Behav.

[10] Wood RT, Griffiths MD (2008). Why Swedish people play online poker and factors that can increase or decrease trust in poker websites: a qualitative investigation. J Gambl Issues..

[11] Forsström D, Jansson-Fröjmark M, Hesser H, Carlbring P. Experiences of Playscan: interviews with users of a responsible gambling tool. Internet Interv. 2017;8:53–62. 10.1016/j.invent.2017.03.003 PMC6096211 30135829

[12] Wohl MJA, Davis CG, Hollingshead SJ. How much have you won or lost? Personalized behavioral feedback about gambling expenditures regulates play. Comput Human Behav. 2017;70:437–45.

[13] Cohen IM, McCormick AV, Davies G. BCLC’s voluntary self-exclusion program from the perspective and experiences of program participants. University of the Fraser Valley. 2017.

[14] Gainsbury S (2012). Responsible gambling strategies. Internet gambling: Current research findings and implications.

[15] Nelson SE, LaPlante DA, Peller AJ, Schumann A, LaBrie RA, Shaffer HJ (2008). Real limits in the virtual world: self-limiting behavior of internet gamblers. J Gambl Stud.

[16] Forsström D, Hesser H, Carlbring P. Usage of a responsible gambling tool: a descriptive analysis and latent class analysis of user behavior. J Gambl Stud. 2016;32(3):889–904. 10.1007/s10899-015-9590-6 26753878

[17] Bernhard BJ, Lucas AF, Dongsuk J (2006). Responsible gaming device research report.

[18] Nova Scotia player card research project (2007). Stage III research report.

[19] Lucar C, Wiebe J, Philander KS (2013). Monetary limits tools for internet gamblers: a review of their availability.

[20] Gold J, Lim MS, Hellard ME, Hocking JS, Keogh L (2010). What’s in a message? Delivering sexual health promotion to young people in Australia via text messaging. BMC Public Health.

[21] Haug S, Kowatsch T, Castro RP, Filler A, Schaub MP (2014). Efficacy of a web- and text messaging-based intervention to reduce problem drinking in young people: study protocol of a cluster-randomised controlled trial. BMC Public Health.

[22] Kerr DA, Pollard CM, Howat P, Delp EJ, Pickering M, Kerr KR (2012). Connecting health and technology (CHAT): protocol of a randomized controlled trial to improve nutrition behaviours using mobile devices and tailored text messaging in young adults. BMC Public Health.

[23] Argo JJ, Main KJ (2004). Meta-analyses of the effectiveness of warning labels. J Public Policy Mark.

[24] Cox EP, Wogalter MS, Stokes SL, Tipton Murff EJ (1997). Do product warnings increase safe behavior? A meta-analysis. J Public Policy Mark.

[25] Wogalter MS (2006). Purposes and scope of warnings. Handbook of warnings.

[26] Rothman AJ, Kelly KM, Hertel AW, Salovey P (2003). Message frames and illness representations: implications for interventions to promote and sustain healthy behavior. The self-regulation of health and illness behaviour.

[27] Rothman AJ, Salovey P (1997). Shaping perceptions to motivate healthy behavior: the role of message framing. Psychol Bull.

[28] Rothman AJ, Stark E, Salovey P (2006). Using message framing to promote healthy behavior: a guide to best practices. Best practices in the behavioral management of chronic diseases.

[29] Blaszczynski A, Ladouceur R, Shaffer HJ (2004). A science-based framework for responsible gambling: the Reno model. J Gambl Stud.

[30] Blaszczynski A, Nower L. A pathways model of problem and pathological gambling. Addiction. 2002;97(5):487–99. 10.1046/j.1360-0443.2002.00015.x 12033650

[31] Sharpe L (2002). A reformulated cognitive–behavioral model of problem gambling: a biopsychosocial perspective. Clin Psychol Rev.

[32] Benhsain K, Taillefer A, Ladouceur R (2004). Awareness of independence of events and erroneous perceptions while gambling. Addict Behav.

[33] Monaghan S (2008). Review of pop-up messages on electronic gaming machines as a proposed responsible gambling strategy. Int J Ment Health Addict..

[34] Monaghan S, Blaszczynski A, Nower L (2009). Do warning signs on electronic gaming machines influence irrational cognitions?. Psychol Rep.

[35] Steenbergh TA, Whelan JP, Meyers AW, May RK, Floyd K (2004). Impact of warning and brief intervention messages on knowledge of gambling risk, irrational beliefs and behaviour. Int Gambl Stud.

[36] Williams RJ, Connolly D (2006). Does learning about the mathematics of gambling change gambling behavior?. Psychol Addict Behav.

[37] Monaghan S, Blaszczynski A (2007). Recall of electronic gaming machine signs: a static versus a dynamic mode of presentation. J Gambl Issues.

[38] Monaghan S, Blaszczynski A (2010). Impact of mode of display and message content of responsible gambling signs for electronic gaming machines on regular gamblers. J Gambl Stud.

[39] Tversky A, Kahneman D (1974). Judgment under uncertainty: Heuristics and biases. Science (80- ).

[40] Leventhal H (1970). Findings and theory in the study of fear communications. Adv Exp Soc Psychol.

[41] Broda A, LaPlante DA, Nelson SE, LaBrie RA, Bosworth LB, Shaffer HJ. Virtual harm reduction efforts for internet gambling: effects of deposit limits on actual internet sports gambling behavior. Harm Reduct J. 2008;5(27). 10.1186/1477-7517-5-27 PMC2519063 18684323

[42] Strahan EJ, White K, Fong GT, Fabrigar LR, Zanna MP, Cameron R (2002). Enhancing the effectiveness of tobacco package warning labels: a social psychological perspective. Tob Control.

[43] Fukunaga R, Bogg T, Finn PR, Brown JW (2013). Decisions during negatively-framed messages yield smaller risk-aversion-related brain activation in substance-dependent individuals. Psychol Addict Behav.

[44] Krawitz A, Fukunaga R, Brown JW (2010). Anterior insula activity predicts the influence of positively framed messages on decision making. Cogn Affect Behav Neurosci.

[45] Rothman AJ, Bartels RD, Wlaschin J, Salovey P (2006). The strategic use of gain-and loss-framed messages to promote healthy behavior: how theory can inform practice. J Commun.

[46] Wansink B, Pope L (2014). When do gain-framed health messages work better than fear appeals?. Nutr Rev.

[47] Glock S, Müller BC, Krolak-Schwerdt S (2013). Implicit associations and compensatory health beliefs in smokers: exploring their role for behaviour and their change through warning labels. Br J Health Psychol.

[48] Hoch SJ, Deighton J (1989). Managing what consumers learn from experience. J Mark.

[49] Levin IP, Chapman DP, Johnson RD (1988). Confidence in judgments based on incomplete information: an investigation using both hypothetical and real gambles. J Behav Decis Mak.

[50] Mussweiler T, Neumann R (2000). Sources of mental contamination: comparing the effects of self-generated versus externally provided primes. J Exp Soc Psychol.

[51] Kardes FR, Kim J, Lim JS (1994). Moderating effects of prior knowledge on the perceived diagnosticity of beliefs derived from implicit versus explicit product claims. J Bus Res.

[52] Gainsbury S, Aro D, Ball D, Tobar C, Russell A (2015). Optimal content for warning messages to enhance consumer decision making and reduce problem gambling. J Bus Res.

[53] Wright P (1979). Concrete action plans in TV messages to increase reading of drug warnings. J Consum Res.

[54] Guillaumier A, Bonevski B, Paul C (2014). Tobacco health warning messages on plain cigarette packs and in television campaigns: a qualitative study with Australian socioeconomically disadvantaged smokers. Health Educ Res.

[55] Vallone DM, Duke JC, Mowery PD, McCausland KL, Xiao H, Costantino JC (2010). The impact of EX®: results from a pilot smoking-cessation media campaign. Am J Prev Med.

[56] Vallone DM, Niederdeppe J, Richardson AK, Patwardhan P, Niaura R, Cullen J (2011). A national mass media smoking cessation campaign: effects by race/ethnicity and education. Am J Health Promot.

[57] Matulewicz N (2015). How do players use a responsible gambling tool? Why user interface design matters.

[58] Yank V, Tribett E, Green L, Pettis J (2015). Learning from marketing: rapid development of medication messages that engage patients. Patient Educ Couns.

[59] Hing N (2003). An assessment of member awareness, perceived adequacy and perceived effectiveness of responsible gambling strategies in Sydney clubs.

[60] Schrans T, Grace J, Schellinck T (2004). 2003 NS VL responsible gaming features evaluation: final report.

[61] Hadden SG (1991). Regulating product risks through consumer information. J Soc Issues.

[62] Wogalter H, Dejoy D, Laughery KR. Warnings and risk communication. London: Taylor & Francis; 1999.

[63] Harris A, Griffiths MD. A critical review of the harm-minimisation tools available for electronic gambling. J Gambl Stud. 2017;33(1):187–221. 10.1007/s10899-016-9624-8 PMC5323476 27289237

[64] Gainsbury S (2011). Player account-based gambling: potentials for behaviour-based research methodologies. Int Gambl Stud.

[65] Bennett GG, Glasgow RE (2009). The delivery of public health interventions via the internet: actualizing their potential. Annu Rev Public Health.

[66] Cunningham JA, Sdao-Jarvie K, Koski-Jännes A, Breslin FC (2001). Using self-help materials to motivate change at assessment for alcohol treatment. J Subst Abus Treat.

[67] Wood RT, Williams RJ (2009). Internet gambling: Prevalence, patterns, problems and policy options.

[68] Bull FC, Kreuter MW, Scharff DP (1999). Effects of tailored, personalized and general health messages on physical activity. Patient Educ Couns.

[69] Carbonneau R, Vitaro F, Brendgen M, Tremblay RE (2015). Variety of gambling activities from adolescence to age 30 and association with gambling problems: a 15-year longitudinal study of a general population sample. Addiction.

[70] Volberg RA, Gupta R, Griffiths MD, Ólason DT, Delfabbro P (2010). An international perspective on youth gambling prevalence studies. Int J Adolesc Med Health.

[71] Blinn-Pike L, Worthy SL, Jonkman JN (2010). Adolescent gambling: a review of an emerging field of research. J Adolesc Health.

[72] Rogers WA, Lamson N, Rousseau GK (2000). Warning research: an integrative perspective. Hum Factors.

[73] Fischer PM, Krugman DM, Fletcher JE, Fox RJ, Rojas TH (1993). An evaluation of health warnings in cigarette advertisements using standard market research methods: what does it mean to warn?. Tob Control.

[74] Fox RJ, Krugman DM, Fletcher JE, Fischer PM (1998). Adolescents’ attention to beer and cigarette print ads and associated product warnings. J Advert.

[75] Leventhal H, Glynn K, Fleming R (1987). Is the smoking decision an “informed choice”? Effect of smoking risk factors on smoking beliefs. JAMA.

[76] Mazanov J, Byrne D (2007). Changes in adolescent smoking behaviour and knowledge of health consequences of smoking. Aust J Psychol.

[77] Hardoon K, Derevensky JL, Gupta R (2003). Empirical measures vs. perceived gambling severity among youth: Why adolescent problem gamblers fail to seek treatment. Addict Behav.

[78] Delfabbro PH, Winefield AH (2000). Predictors of irrational thinking in regular slot machine gamblers. J Psychol.

[79] Dowling NA, Smith D, Thomas T (2005). Electronic gaming machines: are they the ‘crack-cocaine’ of gambling?. Addiction.

[80] Gupta R, Derevensky JL (2000). Adolescents with gambling problems: from research to treatment. J Gambl Stud.

[81] Arthur D, Quester P (2004). Who’s afraid of that ad? Applying segmentation to the protection motivation model. Psychol Mark.

[82] Ho R (1998). The intention to give up smoking: disease versus social dimensions. J Soc Psychol.

[83] Schoenbachler DD, Whittler TE (1996). Adolescent processing of social and physical threat communications. J Advert.

[84] Canada S (2014). Canadian community health survey. Cycle.

[85] Stitt BG, Giacopassi D, Nichols M (2003). Gambling among older adults: a comparative analysis. Exp Aging Res.

[86] Tirachaimongkol LC, Jackson AC, Tomnay JE (2010). Pathways to problem gambling in seniors. J Gerontol Soc Work.

[87] Tira C, Jackson AC, Tomnay JE (2014). Pathways to late-life problematic gambling in seniors: a grounded theory approach. Gerontologist.

[88] Philippe F, Vallerand RJ (2007). Prevalence rates of gambling problems in Montreal, Canada: a look at old adults and the role of passion. J Gambl Stud.

[89] Lewis RA, Reiley DH (2014). Advertising effectively influences older users: how field experiments can improve measurement and targeting. Rev Ind Organ.

[90] Bjerg O (2010). Problem gambling in poker: money, rationality and control in a skill-based social game. Int Gambl Stud.

[91] Abarbanel B, Bernhard B, Singh AK, Lucas A (2015). Impact of virtual atmospherics and functional qualities on the online gambler’s experience. Behav Inf Technol.

[92] Gainsbury S, Suhonen N, Saastamoinen J (2014). Chasing losses in online poker and casino games: characteristics and game play of internet gamblers at risk of disordered gambling. Psychiatry Res.

[93] Auer M, Griffiths MD (2013). Voluntary limit setting and player choice in most intense online gamblers: an empirical study of gambling behaviour. J Gambl Stud.

[94] Afifi TO, LaPlante DA, Taillieu TL, Dowd D, Shaffer HJ (2014). Gambling involvement: considering frequency of play and the moderating effects of gender and age. Int J Ment Health Addict.

[95] Currie SR, Hodgins DC, Wang J, El-Guebaly N, Wynne H, Chen S (2006). Risk of harm among gamblers in the general population as a function of level of participation in gambling activities. Addiction.

[96] Hodgins DC, Schopflocher DP, Martin CR, El-Guebaly N, Casey DM, Currie SR (2012). Disordered gambling among higher-frequency gamblers: who is at risk?. Psychol Med.

[97] Braverman J, Shaffer HJ (2010). How do gamblers start gambling: identifying behavioural markers for high-risk internet gambling. Eur J Pub Health.

[98] LaPlante D, Nelson S, and Gray H. *Breadth and depth involvement: Understanding internet gambling involvement and its relationship to gambling problems*. Psychol Addict Behav. 2014;28(2):396–403. 10.1037/a0033810 23915365

[99] Marshall C, Rossman GB (2011). Designing qualitative research.

[100] Creswell JW (2003). Research design: qualitative, quantitative and mixed methods approaches.

[101] Woodyatt CR, Finneran CA, Stephenson R (2016). In-person versus online focus group discussions: a comparative analysis of data quality. Qual Health Res.

[102] Lune H, Berg BL (2009). Qualitative research methods for the social sciences.

[103] Saldaña J (2016). The coding manual for qualitative researchers.

[104] Shaffer HJ, Hall MN, Vander Bilt J. Estimating the prevalence of disordered gambling behavior in the United States and Canada: a research synthesis. Am J Public Heal. 1999;89(9):1369–76. 10.2105/ajph.89.9.1369 PMC1508762 10474555

[105] Philander K, Gainsbury S (2014). Customised responsible gambling messages.

[106] Holtgraves T (2009). Gambling, gambling activities, and problem gambling. Psychol Addict Behav.

[107] MacKay TL, Bard N, Bowling M, Hodgins D (2014). Do pokers players know how good they are? Accuracy of poker skill estimation in online and offline players. Comput Human Behav..

[108] Barrault S, Varescon I (2013). Cognitive distortions, anxiety, and depression among regular and pathological gambling online poker players. Cyberpsychol Behav Soc Netw..

[109] Hopley AA, Nicki RM (2010). Predictive factors of excessive online poker playing. Cyberpsychol Behav Soc Netw.

[110] McCormack A, Griffiths M (2012). What differentiates professional poker players from recreational poker players? A qualitative study. Int J Ment Health Addict.

[111] Ladouceur R, Blaszczynski A, Lalande DR (2012). Pre-commitment in gambling: a review of the empirical evidence. Int Gambl Stud.

