# Supplementary material for: Strategies to customize responsible gambling messages: a review and focus group study
Source: BMC Public Health. 2018 Dec 17;18:1381. doi: 10.1186/s12889-018-6281-0 (PMC6297977; doi:10.1186/s12889-018-6281-0)
Supplement: Supplementary file 1 — Focus Group Moderator Guide for Strategies to Customize Responsible Gambling Messages: A Review and Focus Group Study. Moderator guide information for focus groups conducted, including research background, initial questions, and proposed RG messages for testing. (PDF 112 kb) [file 12889_2018_6281_MOESM1_ESM.pdf]

Additional File 1
Moderator Guide for *Strategies to Customize Responsible Gambling Messages: A Review and Focus Group Study*

General Research Objectives

The background and rationale for this project is that most warning messages are ineffective in producing behavioural change. This is because messages are written to be broad in an attempt to target all product users. Consequentially, messages are overly general and are considered irrelevant by users. Users are typically aware of warning messages, but are not influenced by these in terms of cognitive or behavioural changes. The aim of the targeted messages is to increase the relevance of warning messages to individuals and encourage the behavioural outcome of engaging with responsible gambling tools (e.g., reading information, setting spend or time limits, etc).

The purpose of the focus groups is to review a list of messages intended to encourage users of the PlayNow online gambling system to use the responsible gambling tools available through the site's responsible gambling program, GameSense. We're looking to understand differences between groups of players (e.g., men vs. women or people who gamble frequency vs. people who don't gamble very often) for the purpose of understanding how they would likely respond to the messages. The focus group should clarify that we are not interested in generic perspectives of what the participants think would work for others, but what would work specifically for people like them. The moderator should be clear on this point and redirect if comments start along lines of presumptions of others.

In addition to feedback and review of our suggested messages, we also want to learn more about what words, terms, themes, language, and other new ideas the participants might have. Participants may also create wholly new messages that they consider to be better than those provided to them for discussion.

Respondent Profile

There are four focus groups with different populations:

1. Young adults (age 18-24)
2. Seniors (age 60+)
3. Skill players
4. Frequent gamblers (weekly or more often)

All participants will be screened to ensure that they have gambled online at least once during the prior 12 months.

Interview Location

Online

Introduction

- Explain that the messages are intended to attract players to the PlayNow online gambling site's responsible gambling tools, known as the GameSense program
- Ask focus groups if they are aware of the GameSense tools and information and whether they see any messages about these tools and web pages
- Brief explanation of what the Game Sense tools are:
    - *Player Assessment Quiz* – a tool to complete a self-assessment of your gambling knowledge and behavior
    - *Managing Your Play* – a tool to set personalised limits on time and dollar spend for gambling
    - *Voluntary Self-Exclusion* – a tool that sets a voluntary self-ban on access to the PlayNow system
    - *Dollars & Sense Guide* – a guide to help players understand how various gambling patterns can increase of decrease the costs of their gambling
    - *GameSense Info Centre locations* – A map locator of in-person GameSense Info Centres

Suggested Questions

- Have you seen messages about GameSense and responsible gambling (e.g., information about problems, setting limits)? Why do or do not you respond to these messages?
- What do you consider to be useful or interesting about the GameSense resources?
- What messages would you design to encourage people like you to engage with these tools and resources? What words would you use or avoid? What would make you click on the link in a message?
- What do you think of these messages? Are there any changes you would make so the message is more relevant to you? Would you use different words?

Messages by group

1. Young adults (age 18-24)
    - Do you know how much money you spent this week? Check how you are doing. [link to personal player history]
    - Do you know how much you are spending? Check your play summary. [link to personal player history]
    - Have a moment? Test your gambling habits. [link to self assessment quiz]
    - What kind of player are you? Take this short quiz. [link to self assessment quiz]
    - Play often? Check out these 7 gambling tips [link to tips]
        - Alternative: New to PlayNow? Check out these 7 gambling tips [link to tips]
    - Are you a gambling expert? Test your knowledge of gambling odds. [link to quiz]
    - All players need to stick to their limits. Have you set your spend limit? [link to limit setting tools]
        - Alternative: All players need to stick to their limits. Do you know how much you have spent? [link to limit setting tools]

2. Seniors (age 60+)
    - Do you know how much money you spent this week? Check how you are doing. [link to personal player history]
    - Have you spent more than you can afford? Check your play summary. [link to personal player history]
    - Have a moment? Test your gambling habits. [link to self assessment quiz]
    - What kind of player are you? Take this short quiz. [link to self assessment quiz]
    - Play often? Check out these 7 gambling tips [link to tips]
        - Alternative: New to PlayNow? Check out these 7 gambling tips [link to tips]
    - All players need to stick to their limits. Have you set your spend limit? [link to limit setting tools]
        - Alternative: All players need to stick to their limits. Do you know how much you have spent? [link to limit setting tools]

3. Skill players
    - Do you know how much money you spent this week? Check how you are doing. [link to personal player history]
    - Have you spent more than you can afford? Check your play summary. [link to personal player history]
    - Have a moment? Test your gambling habits. [link to self assessment quiz]
    - What kind of player are you? Take this short quiz. [link to self assessment quiz]
    - Play often? Check out these 7 gambling tips [link to tips]
        - Alternative: New to PlayNow? Check out these 7 gambling tips [link to tips]
    - Are you a gambling expert? Test your knowledge of gambling odds. [link to quiz]
    - All players need to stick to their limits. Have you set your spend limit? [link to limit setting tools]
        - Alternative: All players need to stick to their limits. Do you know how much you have spent? [link to limit setting tools]

4. Frequent gamblers (weekly or more often)
    - Do you know how much money you spent this week? Check how you are doing. [link to personal player history]
    - Have you spent more than you can afford? Check your play summary. [link to personal player history]
    - Have a moment? Test your gambling habits. [link to self assessment quiz]
    - What kind of player are you? Take this short quiz. [link to self assessment quiz]
    - Play often? Check out these 7 gambling tips [link to tips]

- Even frequent players need to stick to their limits. Have you set your spend limit? [link to limit setting tools]
    - Alternative: Even frequent players need to stick to their limits. Do you know how much you have spent? [link to limit setting tools]
